# Hydroxyurea alters hematological, biochemical and inflammatory biomarkers in Brazilian children with SCA: Investigating associations with βS haplotype and α-thalassemia

**DOI:** 10.1371/journal.pone.0218040

**Published:** 2019-07-15

**Authors:** Sètondji Cocou Modeste Alexandre Yahouédéhou, Caroline Conceição da Guarda, Camylla Vilas Boas Figueiredo, Rayra Pereira Santiago, Suellen Pinheiro Carvalho, Luciana Magalhães Fiuza, Uche Samuel Ndidi, Rodrigo Mota Oliveira, Magda Oliveira Seixas Carvalho, Valma Maria Lopes Nascimento, Larissa Carneiro Rocha, Isa Menezes Lyra, Elisângela Vitória Adorno, Marilda Souza Goncalves

**Affiliations:** 1 Laboratório de Investigação em Genética e Hematologia Translacional, Instituto Gonçalo Moniz, Salvador, Bahia, Brasil; 2 Laboratório de Pesquisa em Anemia, Departamento de Análises Clínicas, Faculdade de Farmácia, Universidade Federal da Bahia, Salvador, Bahia, Brasil; 3 Department of Biochemistry, Ahmadu Bello University, Zaria, Nigeria; 4 Hospital Universitário Prof. Edgard Santos, Salvador, Bahia, Brasil; 5 Fundação de Hematologia e Hemoterapia da Bahia, Salvador, Bahia, Brasil; Universidade Nova de Lisboa Instituto de Higiene e Medicina Tropical, PORTUGAL

## Abstract

This study investigated the effects of hydroxyurea (HU) on hematological, biochemical and inflammatory parameters in children with sickle cell anemia (SCA) in association with β^S^ haplotype and α-thalassemia. We included 22 children with SCA who were followed for an average of 14.5 months. Laboratory parameters were assessed by electronic methods, and molecular analysis was investigated by PCR-RFLP and allele-specific PCR. Results showed significant increases in hemoglobin, HbF, hematocrit, MCV, MCH, glucose, HDL-C and albumin levels, as well as significant decreases in MCHC and AST levels, WBC, neutrophils, eosinophils, lymphocytes and reticulocytes, in children during HU therapy. HbF levels were positively correlated with hemoglobin, hematocrit, MCV and total protein, yet negatively correlated with MCHC, RDW, AAT and AST during HU therapy (*p*<0.05). Children who carried the Central African Republic haplotype, in response to HU therapy, presented significant increases in hemoglobin, hematocrit, triglycerides and uric acid levels, as well as significant decreases in MCHC, AST and direct bilirubin levels, WBC, neutrophils, eosinophils, lymphocytes and reticulocytes. Those with the Benin haplotype presented increases in HbF and albumin levels, and a reduction in platelet counts (*p*<0.05). Children with α-thalassemia presented decreased ALT during HU use, while those without this deletion presented increases in hemoglobin, hematocrit, MCV, MCH, HDL-C and albumin, as well as decreases in MCHC, neutrophils, lymphocytes, reticulocytes and AST (*p*<0.05). Hence, regardless of its use in association with β^S^ haplotypes or α-thalassemia, HU seems to be linked to alterations in hemolytic, inflammatory, hepatic, lipid and glycemic profiles.

## Introduction

Sickle cell anemia (SCA) is one of the most common inherited monogenic diseases in the world, characterized by chronic hemolytic anemia, vaso-occlusive events (VOE) and chronic organ injury [1]. Clinical profile and life expectancy vary widely among individuals with SCA, which can be explained by several factors, including genetic modifiers, such as haplotypes linked with the beta S (β^S^)-globin gene cluster and alpha 2 deletion of 3.7 kb thalassemia (α^2 del 3.7kb^ thalassemia) [1]. The Benin (BEN), Bantu or Central African Republic (CAR), Senegal, Cameroon and Arab/Hindu haplotypes identified in the β^S^-globin gene cluster have been associated with variability in fetal hemoglobin (HbF) levels, which is known to be a classic modulator of this disease [2–4]. Likewise, individuals with SCA who are carriers of α-thalassemia present increases in hemoglobin concentrations and red blood cell (RBC) count, as well as decreases in mean corpuscular volume (MCV), mean corpuscular hemoglobin (MCH), mean corpuscular hemoglobin concentration (MCHC), reticulocyte counts and bilirubin levels [5–7]. However, α-thalassemia has also been associated with an elevated frequency of VOE, which may be due to increased hematocrit and, consequently, blood viscosity in individuals with SCA [7].

Among the three agents known to enhance HbF production (Sodium butyrate, 5-azacytidine and hydroxyurea—HU), HU, approved in 1998 by the U.S. Food and Drug Administration (FDA) for the treatment of individuals with clinically severe SCA, remains the most commonly used [3,8]. The main benefit associated with HU therapy is increased HbF levels, which have been directly associated with decreased HbS polymerization, reduced incidence of VOE, frequency and length of hospital stays, blood therapy and acute chest syndrome (ACS). HU therapy also reduces health care costs and increases survival rates [9–12]. Furthermore, studies have demonstrated the association of HU with a reduction in white blood cell (WBC) and reticulocyte counts, as well as bilirubin and lactate dehydrogenase (LDH) levels, in addition to increased MCV [3,4]. Although it is believed that increases in HbF levels mediates HU efficacy among individuals with SCA, it was reported that improvement in patients’ clinical profiles appears prior to any significant increase in their HbF levels, suggesting that HU may modulate other laboratory parameters beside the classical increase seen in HbF [11]. Regarding HbF levels and the patient clinical profile, there is a great variability in response among individuals treated with HU, which may be due to genetic factors, including the β^S^-globin gene cluster haplotype [13,14]. Moreover, despite evidence demonstrating its efficacy, HU is underused in younger individuals with SCA due to a range of issues, mainly side effects, which have not been completely elucidated [15,16].

Therefore, we sought to investigate the wider effect of HU on hematological, biochemical and inflammatory parameters in children with SCA who carry the β^S^ haplotype, with or without α-thalassemia, in a prospective study.

## Materials and methods

### Subjects and ethical aspects

The present prospective study included 22 children with SCA (HbSS). Laboratory parameters were assessed before and during HU treatment, and the median length of HU use was 14.5 months (6 to 72 months). The average age of the children was 8.5±3.4 years (median: 7.5 years) at the beginning of the study and 12 (54.55%) were female. All patients were seen at the outpatient service of the Fundação de Hematologia e Hemoterapia da Bahia (HEMOBA). They reported the prior regular use of folic acid, and, after study recruitment, HU was prescribed at doses lower than its maximum tolerated dose (MTD) and ranging between 10–25 mg/kg/day (median: 15 mg/kg/day). All legal guardians reported that HU treatment was very important to their children, and affirmed that the medication was taken regularly unless ordered to interrupt treatment by a physician, which strongly indicates HU compliance. For inclusion, children were required to be in steady state, characterized as the absence of acute crisis and without the use of blood therapy, for three months prior to blood collection procedures. Children on blood therapy and those with infections were excluded.

This study was approved by the Institutional Review Board of the São Rafael Hospital, and was conducted in accordance with the Declaration of Helsinki and its amendments. In addition, the legal guardians of all children signed a term of informed consent prior to enrollment in the study.

### Laboratory methods

Blood samples were drawn at the time of each individual’s enrollment by venipuncture, in the morning after 12 hours of fasting under standardized conditions. Hematological analyses were performed using a Cell Dyn-Ruby electronic cell counter (Abbott Diagnostics, Wiesbaden, Germany). Qualitative and quantitative hemoglobin profiles were determined by high-performance liquid chromatography (HPLC/Variant II; BIO-RAD, Hercules, CA, USA). Biochemical parameters were measured in serum by an immunochemistry assay using an A25 spectrophotometer analyzer (Biosystems SA, Barcelona, Spain). Inflammatory proteins, such as alpha-1 antitrypsin (AAT) and C-reactive protein (C-RP), were measured by immunochemistry using the Immage 800 System (Beckman Coulter, Fullerton, CA, USA). Serum ferritin was measured by immunoassay using an Access 2 Immunoassay system (Beckman Coulter, Fullerton, CA, USA).

Genomic DNA was extracted from peripheral blood using the Flexigen 250 kit (Qiagen, Hilden, Germany). β^S^ haplotypes were investigated by polymerase chain reaction-restriction fragment length polymorphism (PCR-RFLP) [17], and α^2 del 3.7kb^ thalassemia was assessed by allele-specific PCR [18].

All analyses were performed at the Laboratório de Análises Clínicas and Laboratório de Pesquisa em Anemia, Faculdade de Farmácia, Universidade Federal da Bahia and the Laboratório de Investigação em Genética e Hematologia Translacional, Instituto Gonçalo Moniz–FIOCRUZ/BA.

### Statistical analysis

Statistical analyses were performed using EpiInfo 7.0 and GraphPad Prism v5.0 software, with *p* values below 0.05 considered statistically significant. Distributions of quantitative variables were determined by the Shapiro-Wilk test. The mean values of variables (laboratory parameters), measured before and during HU therapy, were compared using the paired t-test for normal distribution, and Wilcoxon’s test for non-normal distribution. Results were expressed as mean ± standard deviation. Pearson’s correlation coefficient analysis was performed to assess the strength of linear relationships between two quantitative variables with normal distribution.

## Results

### Laboratory parameters of children with SCA before and during HU therapy

The laboratory parameters of the SCA children before and during HU therapy are shown in Table 1, and S1 Fig. After the onset of HU therapy, hematological analyses showed significant increases in mean HbF, hemoglobin, hematocrit, MCV and MCH, in addition to significant reductions in reticulocytes and MCHC (*p*<0.05). In all, 14 out of 22 children (63.64%) presented increases in HbF levels during HU therapy. Furthermore, all children had significant decreases in WBC, neutrophils, eosinophils and lymphocytes during HU therapy (*p*<0.05). Biochemical analysis revealed elevated glucose, HDL-C and albumin, as well as decreased aspartate aminotransferase (AST), in the children following the introduction of HU therapy (*p*<0.05).

**Table 1:** Laboratory parameters of SCA children, before and during HU therapy.

	Before HU, N = 22 M ± SD	During HU, N = 22 M ± SD	*p* value
**Hemoglobin**			
HbF, %	9.95±6.28	12.72±5.77	0.04**
HbS, %	86.52±6.05	84.87±5.50	0.18*
**Erythrogram**			
RBC, x10^9^/mL	2,74±0,63	2,74±0,57	0.89*
Hemoglobin, g/dL	8.15±1.30	8.77±1.24	0.01*
Hematocrit, %	23.41±3.92	26.11±4.07	<0.01*
MCV, fL	86.67±9.03	96.72±10.26	<0.01*
MCH, pg	30.25±3.45	32.59±3.47	<0.01*
MCHC, %	34.90±1.48	33.70±0.94	<0.01*
Erythroblast (/10^2^ leukocytes)	2.50±1.92	0.86±0.88	<0.01**
**Leukogram**			
WBC, /mL	15283±3931	10772±3547	<0.01*
Neutrophil, /mL	7275±2279	5174±2409	<0.01*
Eosinophil, /mL	1097±816	508±439	<0.01**
Lymphocyte, /mL	5717±2576	3958±1628	<0.01*
Monocyte, mL	950±468	989±533	0.99**
**Platelets**			
Platelet, x10^3^/mL	433±105	400±140	0.26*
**Hemolysis**			
Total bilirubin, mg/dL	2.54±1.77	2.69±1.65	0.30**
Direct bilirubin, mg/dL	0.54±0.29	0.43±0.24	0.13**
Indirect bilirubin, mg/dL	1.99±1.54	2.26±1.62	0.24**
Lactate dehydrogenase, U/L	1072±431.8	1026±375.3	0.51*
Reticulocyte, %	8.01±3.17	4.56±1.66	<0.01*
**Lipids and glucose**			
Total cholesterol, mg/dL	120.0±26.0	124.7±29.6	0.28*
HDL-C, mg/dL	31.73±7.45	40.42±12.66	<0.01**
LDL-C, mg/dL	69.96±19.69	64.35±23.62	0.20**
Triglycerides, mg/dL	92.23±51.44	106.5±55.65	0.05**
Glucose, mg/dL	71.00±9.35	83.86±9.58	<0.01*
**Renal panel**			
Urea, mg/dL	18.86±7.48	21.04±8.85	0.43**
Creatinine, mg/dL	1.68±1.76	0.85±1.09	0.47**
**Hepatic panel**			
Aspartate aminotransferase, U/L	57.50±17.97	44.32±16.50	<0.01*
Alanine aminotransferase, U/L	24.05±11.39	22.86±12.04	0.44**
Total protein, g/dL	7.84±0.70	8.17±0.77	0.23**
Albumin, g/dL	4.34±0.34	4.73±0.32	<0.01*
**Inflammatory markers**			
Uric acid, mg/dL	2.48±1.72	3.41±1.77	0.07*
Ferritin, ng/dL	501.6±625.6	611.5±791.0	0.51**
C-reactive protein, mg/L	7.30±7.17	7.35±12.62	0.40**
Alpha 1 antitrypsin, mg/dL	168.3±39.98	104.2±47.41	<0.01**

RBC: red blood cell, MCH: mean corpuscular hemoglobin, MCV: mean corpuscular volume, MCHC: mean corpuscular hemoglobin concentration, HbS: S hemoglobin, HbF: Fetal hemoglobin, WBC: white blood cell, HDL-C: high-density lipoprotein cholesterol, LDL-C: low-density lipoprotein cholesterol, VLDL-C: very low-density lipoprotein cholesterol, M ± SD: mean ± standard deviation, N: number of individuals

*Paired T-test

** Wilcoxon test

### Correlations between HbF and laboratory parameters during HU therapy

**Fig 1:**
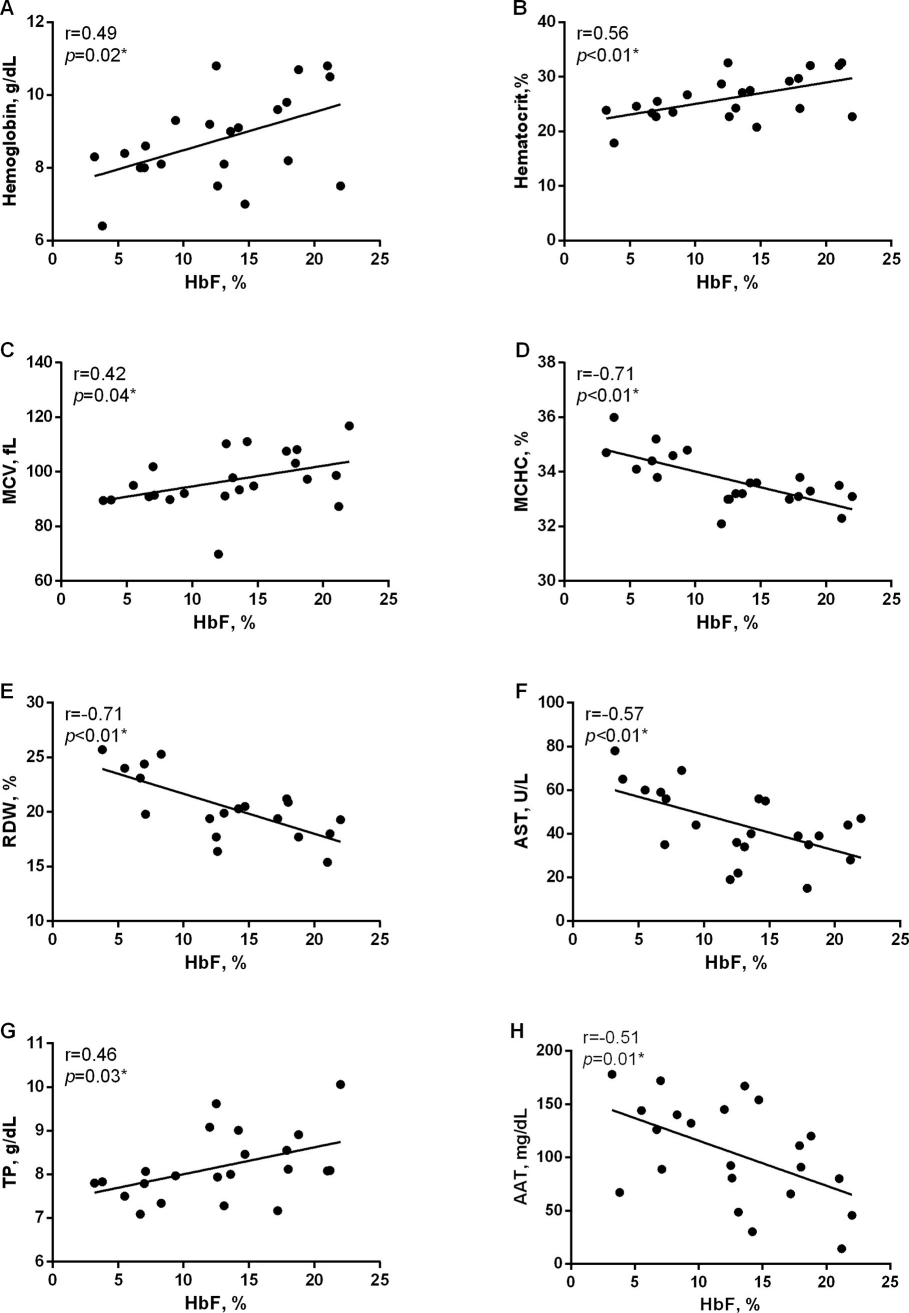
Correlations between HbF levels and hematological and biochemical parameters in children with SCA during HU therapy. HbF levels were positively correlated with hemoglobin (**A**), hematocrit (**B**), MCV (**C**) and TP (**G**), while negatively correlated with MHCH (**D**), RDW (**E**), AST (**F**) and AAT (**H**). MCV: mean corpuscular volume, MCHC: mean corpuscular hemoglobin concentration, RDW: red cell distribution width, AST: aspartate aminotransferase, TP: total protein, AAT: alpha 1 antitrypsin, *Pearson’s correlation.

Fig 1 shows the results of correlation analysis in SCA children undergoing HU therapy. HbF levels were found to be positively correlated with hemoglobin, hematocrit, MCV and total protein levels, yet negatively correlated with MCHC, RDW, AST and AAT (*p*<0.05). When performing correlation analysis in the children prior to the onset of HU therapy, no positive or negative associations were observed.

### Laboratory parameters of children with SCA before and during HU therapy according to β^S^ haplotypes and α-thalassemia

The distribution analyses of β^S^ haplotypes and α^2 del 3.7kb^ thalassemia showed that 45% were carriers of the CAR/CAR genotype, while 35% and 20% were CAR/BEN and BEN/BEN, respectively. Fourteen children (77.78%) presented the αα/αα genotype (without α-thalassemia) and 22.22% the -α/αα or -α/-α genotypes (with α-thalassemia).

As it is known that the response to HU can be affected by β^S^ haplotypes and α-thalassemia, we performed an association analysis using a dominant genetic model comparing all laboratory parameters of the children prior to and during HU therapy. This analysis showed that children with the CAR haplotype presented significant increases in hemoglobin, hematocrit, MCV, MCH, glucose, triglycerides, uric acid and albumin levels, as well as significant reductions in MCHC, AST, direct bilirubin and AAT levels, as well as WBC, neutrophils, eosinophils, lymphocytes and reticulocytes during HU therapy. Children who were carriers of the BEN haplotype presented elevated HbF, MCV, MCH, glucose and albumin levels, in addition to reduced platelets during HU therapy (*p*<0.05) (Table 2).

**Table 2:** Laboratory parameters of children with SCA according to β^S^ haplotype before and during HU therapy.

	CAR/CAR + CAR/BEN, n = 16			BEN/BEN, n = 4		
Parameter	Before HU	During HU	*p* value	Before HU	During HU	*p* value
HbF, %	10.57±6.68	12.14±5.46	0.27*	9.65±5.84	17.42±4.77	<0.01*
Hemoglobin, g/dL	8.12±1.26	8.68±1.16	0.03*	8.32±1.62	9.55±1.58	0.15*
Hematocrit, %	23.19±3.79	25.81±3.98	<0.01*	24.13±5.38	28.73±4.63	0.07*
MCV, Fl	85.93±9.29	94.39±9.11	<0.01*	87.30±10.32	104.45±11.62	<0.01*
MCH, pg	30.13±3.39	31.90±3.29	<0.01**	30.43±5.00	34.78±3.95	<0.01*
MCHC, %	35.08±1.39	33.78±1.04	<0.01*	34.68±1.88	33.30±0.29	0.50*
WBC, /mL	16004±3064	11149±2717	<0.01*	10668±4978	6550±2646	0.14*
Neutrophils, /mL	7859±2064	5108±2035	<0.01*	5161±2510	3288±1623	0.11*
Eosinophils, /mL	1186±836	600±459	<0.01*	959±957	114±62	0.12**
Lymphocytes, /mL	5647±2181	4284±1653	0.02*	3832±2049	2444±992	0.20*
Platelets, x10^3^/mL	427±87	430±131	0.92*	363±83	250±107	0.03*
Reticulocytes, %	8.07±3.39	4.79±1.71	<0.01*	7.02±3.10	3.75±0.46	0.25**
Glucose, mg/dL	71.06±9.05	84.50±7.92	<0.01*	76.25±9.21	88.00±13.52	0.01*
Triglycerides, mg/dL	92.94±57.96	116.2±62.59	0.01*	101.8±33.08	84.25±11.44	0.31*
Uric acid, mg/dL	2.45±1.78	3.63±1.88	0.03**	2.97±1.54	3.41±0.84	0.70*
AST, U/L	61.00±17.60	43.25±16.03	<0.01*	49.00±17.61	45.75±8.26	0.58*
Direct bilirubin, mg/dL	0.58±0.31	0.40±0.19	0.01**	0.37±0.22	0.44±0.18	0.28*
Albumin, g/dL	4.40±0.35	4.68±0.30	<0.01*	4.32±0.30	5.0±0.36	0.02*
Alpha 1 antitrypsin, mg/dL	165.9±43.19	111.6±45.23	<0.01**	167±33.20	62.08±29.05	0.01*

HbF: Fetal hemoglobin, MCH: mean corpuscular hemoglobin, MCV: mean corpuscular volume, MCHC: mean corpuscular hemoglobin concentration, WBC: white blood cell, AST: aspartate aminotransferase, n: number of individuals

*Paired T test

** Wilcoxon test, Data presented as means ± standard deviation

Regarding α-thalassemia, during HU therapy the children without α-thalassemia had elevated hemoglobin, hematocrit, MCV, MCH, glucose, HDL-C and albumin levels, in addition to decreased MCHC and AST, as well as WBC, neutrophils, eosinophils, lymphocytes and reticulocytes (*p*<0.05). Those with α-thalassemia presented significantly increased glucose levels and decreased WBC and eosinophils, as well as lower alanine aminotransferase (ALT) levels, during HU therapy (*p*<0.05) (Table 3).

**Table 3:** Laboratory parameters of children with SCA according to α^2del 3.7 kb^ thalassemia before and during HU therapy.

	αα/αα, n = 14			-α/αα and -α/-α, n = 4		
Parameter	Before HU	During HU	*p* value	Before HU	During HU	*p* value
HbF, %	10.54±6.82	13.26±5.73	0.12*	8.61±5.91	9.57±3.10	0.71*
Hemoglobin, g/dL	7.81±1.13	8.52±1.17	<0.01*	8.35±0.94	9.15±1.20	0.39*
Hematocrit, %	22.21±3.64	25.21±3.77	<0.01*	24.53±2.66	27.55±4.01	0.30*
MCV, Fl	89.68±7.44	100.00±8.41	<0.01*	77.13±10.24	85.84±10.69	0.07*
MCH, pg	31.65±3.03	33.87±2.47	<0.01*	26.20±2.82	28.67±4.21	0.18*
MCHC, %	35.28±1.52	33.91±0.91	<0.01**	34.05±1.35	33.32±0.99	0.54*
WBC, /Ml	15952±3534	10963±3091	<0.01*	10300±3339	7675±2384	0.04*
Neutrophils, /mL	7716±2103	4766±1956	<0.01*	5061±1882	3792±1499	0.08*
Eosinophils, /mL	1250±961	607±484	0.01**	1074±381	328±316	<0.01*
Lymphocytes, /mL	5648±2196	4401±1770	0.04*	3493±1134	2815±947	0.27*
Reticulocytes, %	7.75±2.78	4.51±1.27	<0.01*	7.32±5.64	3.55±1.47	0.17*
Glucose, mg/dL	72±7.74	83.07±7.29	<0.01**	75.50±8.58	91.50±14.20	0.04*
HDL-C, mg/dL	31.14±7.94	37.50±9.39	0.02**	30.50±6.56	52.30±21.95	0.18*
AST, U/L	61.00±15.97	45.57±14.43	0.01*	51.50±25.25	42.50±18.70	0.24*
ALT, U/L	25.36±12.18	26.50±12.84	0.82**	26.75±12.50	16.25±6.24	0.04*
Albumin, g/dL	4.30±0.33	4.69±0.30	<0.01*	4.47±0.26	4.82±0.52	0.24*

HbF: Fetal hemoglobin, MCH: mean corpuscular hemoglobin, MCV: mean corpuscular volume, MCHC: mean corpuscular hemoglobin concentration, WBC: white blood cell, AST: aspartate aminotransferase, ALT: alanine aminotransferase, n: number of individuals

*Paired T test

** Wilcoxon test, Data presented as means ± standard deviation

## Discussion

The present study investigated the wider effect of HU on laboratory parameters in children with SCA, according to the presence of the β^S^ haplotype and α-thalassemia. It has been reported that individuals with SCA taking HU may present an improved clinical profile before significant increases in HbF levels are seen, and it is known that HU response can vary according to β^S^ haplotype and α-thalassemia [11,13,14].

As expected, HU intake resulted in a significant increase in HbF levels, which was observed in 14 (63.64%) children. This finding corroborates data from previous studies demonstrating that HU use was associated with higher HbF levels [19–22]. Moreover, increases in HbF were observed in some individuals [23–25], reinforcing the notion that not all individuals with SCA or SCD respond to HU therapy by initially presenting increased HbF levels. Additionally, HU was associated with higher hemoglobin, hematocrit, MCV and MCH, as well as lower MCHC and reticulocyte and erythroblast counts. These findings, also reported by previous studies [16,22,25–27], serve to confirm the association between HU therapy and an improvement in the hemolytic profile of individuals with SCA.

The cytoreductive effect of HU, demonstrated by significant reductions in WBC, neutrophils, eosinophils and lymphocytes, is supported by other studies that also reported similar results [16,22,27,28]. In addition, we observed a significant decrease in levels of the anti-inflammatory protein AAT during HU therapy. These findings suggest an association between HU use and reduced WBC counts mediated and/or followed by decreases in AAT levels and, consequently, improvement in the inflammatory state generally presented by individuals with SCA [26].

In individuals with SCA, the anti-inflammatory role of HDL-C changes to a pro-inflammatory one when bound to free hemoglobin released during hemolysis; this has been associated with endothelial injury and worsening of the inflammatory profile [29,30]. In the present study, children also presented significant increases in HDL-C levels during HU therapy, with eight (36.36%) presenting HDL-C levels above reference values. This finding demonstrates the beneficial effect of HU on cholesterol, and corroborates data from our recent study showing an association between HU use and variation in cholesterol concentrations in individuals with SCA [3]. A previous study also showed that HU modulates the abnormalities of RBC membrane fatty acid composition, which in addition to the vasodilators, nitric oxide and Prostaglandin E2 generated by HU, may lead to clinical improvements prior to increases in HbF induced by HU therapy [31]. Surprisingly, the children also presented increases in glucose levels during HU therapy, although these remained within normal clinical ranges. This increase may be due to the changes observed in cholesterol concentrations in children during HU therapy, since it is known that glucose and lipid metabolism are linked to each other and that hypertriglyceridemia and low HDL-C may be the cause as well as the consequence of hyperglycemia [32]. In addition, significant decreases in AST levels and increased albumin were seen during the use of HU. Contrary to this finding, Colombatti and colleagues observed a significant reduction in AST post-HU therapy [22], which may be due to differences in the clinical profiles of individuals in the respective study groups. Based on their findings, Ragg and colleagues suggested that, in addition to increased HbF observed in individuals with SCD treated by HU, some clinical improvements may be the result of a reduced imbalance in serum protein levels [33].

HbF levels were positively correlated with hemoglobin, hematocrit, MCV and total protein levels, in addition to negatively correlated with MCHC, RDW, as well as AST and AAT levels. A previous study demonstrated a negative correlation between HbF levels and hemolytic biomarkers, reticulocytes and LDH levels, in individuals with SCA [34]. Although we did not find this correlation, our results also suggest that the increases in HbF levels induced by HU are followed by improvements in the hemolytic and inflammatory profiles of the children with SCA studied herein. Furthermore, these findings suggest that, in addition to inducing HbF synthesis, HU can also alter metabolic and hepatic biomarkers by way of yet unknown direct or indirect mechanisms. A recent study performed in individuals with β-thalassemia demonstrated the effects of HU on the metabolic profile [35]. Previous studies also reported the effectiveness of HU for the treatment and prevention of proteinuria in individuals with SCD [36–38]. Nonetheless, further follow-up studies including larger numbers of individuals with SCA that evaluate HU dosage and length of use are needed to confirm the associations seen between HU and metabolic and hepatic biomarkers, as well as to investigate the clinical impact of these effects.

Regarding the distribution of haplotypes, 45% of the children were carriers of CAR/CAR genotype, while 35% and 20% had CAR/BEN and BEN/BEN genotypes, respectively, which corroborates previously reported frequencies in Brazilian individuals with SCA [13]. Our analysis of laboratory parameters, using the dominant genetic model, in children before and during HU therapy found significantly increased HbF levels only in carriers of the BEN haplotype on HU, which corroborates the results of a previous study [39]. These authors suggested that this may be explained by the high frequency of the favorable polymorphism *BCL11A* rs1427407 in these individuals. In the present study, HU was also associated with significant decreases in platelet counts in children with the BEN haplotype. In addition, children with the CAR haplotype presented significantly increased hemoglobin, hematocrit, triglycerides and uric acid, as well as significantly decreased MCHC, WBC, neutrophils, eosinophils, lymphocytes, reticulocytes, AST and direct bilirubin during HU therapy, which is suggestive of improvements in hemolytic, inflammatory and hepatic biomarkers. Regardless of haplotype, and in addition to being associated with significant increases in MCV and MCH, as well as significant decreases in AAT, HU therapy was also found to be associated with significantly increased levels of glucose and albumin.

Distribution analysis of α^2 del 3.7kb^ thalassemia showed similar frequencies to those reported by Adorno and colleagues [40]. Using the dominant genetic model, laboratory parameter analysis in children before and during HU therapy revealed significantly decreased ALT during HU therapy in carriers of α-thalassemia, while those without α-thalassemia presented significant increases in hemoglobin, hematocrit, MCV, MCH, HDL-c and albumin, as well as significant decreases in MCHC, neutrophils, reticulocytes and AST. These findings corroborate previous studies suggesting that α-thalassemia attenuates the effect of HU [41–43], in addition to promoting a beneficial response to HU in SCA individuals without α-thalassemia. Contrary to our findings, other studies found significantly increased hemoglobin, hematocrit, MCV, MCH and HbF, as well as significantly decreased HbS, bilirubin and reticulocytes in SCA individuals with α-thalassemia on HU [25,44]. This discrepancy may be due to other intrinsic genetic factors in the individuals enrolled in the respective study groups. Our data further corroborate an association between HU and decreased WBC and eosinophils that was also reported by a previous study [7]. However, we discovered increased glucose levels, regardless of the presence or absence of α-thalassemia.

## Conclusion

The results of the present study confirm the association between HU therapy and higher HbF levels and suggest that exclusively focusing on HbF levels may not be the most suitable method of assessing HU response in children with SCA. Due to increases in HbF levels and/or via parallel pathways, HU therapy, whether in association or not with β^S^ haplotypes and α-thalassemia, is positively correlated with improvements in hemolytic and inflammatory profiles. Unexpectedly, our results also suggest that, at a dose under MTD, HU may also affect metabolic biomarkers, since it was found to be associated with changes in glucose, total protein, albumin, AST and HDL-C. The principal limitation of the present study is its relatively small sample size. Accordingly, the effects on metabolic biomarkers linked to HU therapy reported herein deserve further scrutiny in a larger study population.

## Supporting information

S1 Fig Laboratory parameters of SCA children, before and during HU therapy. All data presented statistical significant difference. MCH: mean corpuscular hemoglobin, MCV: mean corpuscular volume, MCHC: mean corpuscular hemoglobin concentration, HbF: Fetal hemoglobin, WBC: white blood cell, HDL-C: high-density lipoprotein cholesterol, AST: Aspartate aminotransferase, AAT: Alpha 1 antitrypsin.
(DOCX)

S1 File Database. (XLSX)

## References

[1] Kato GJ, Piel FB, Reid CD, Gaston MH, Ohene-Frempong K, Krishnamurti L, et al Sickle cell disease. Nat Rev Dis Primer. 2018 3 15;4(18010):1–22. 10.1038/nrdp.2018.10 29542687

[2] Fong C, Lizarralde-Iragorri MA, Rojas-Gallardo D, Barreto G. Frequency and origin of haplotypes associated with the beta-globin gene cluster in individuals with trait and sickle cell anemia in the Atlantic and Pacific coastal regions of Colombia. Genet Mol Biol. 2013;36(4):494–7. 10.1590/S1415-47572013000400005 24385850 PMC3873178

[3] Yahouédéhou SCMA Carvalho MOS, Oliveira RM Santiago RP, da Guarda CC Carvalho SP, et al Sickle Cell Anemia Patients in Use of Hydroxyurea: Association between Polymorphisms in Genes Encoding Metabolizing Drug Enzymes and Laboratory Parameters. Dis Markers. 2018;2018a:1–11. 10.1155/2018/6105691 PMC5829363 29619129

[4] Aleluia MM, Santiago RP, da Guarda CC, Fonseca TCC, Neves FI, Quinto RS, et al Genetic modulation of fetal hemoglobin in hydroxyurea-treated sickle cell anemia: Aleluia et al. Am J Hematol. 2017 5;92(5):E70–E72. 10.1002/ajh.24680 28195442 PMC5389903

[5] Steinberg MH. Genetic Etiologies for Phenotypic Diversity in Sickle Cell Anemia. Sci World J. 2009;9:46–67. 10.1100/tsw.2009.10 PMC5823205 19151898

[6] Camilo-Araújo RF, Amancio OMS, Figueiredo MS, Cabanãs-Pedro AC, Braga JAP. Molecular analysis and association with clinical and laboratory manifestations in children with sickle cell anemia. Rev Bras Hematol E Hemoter. 2014 9;36(5):334–9. 10.1016/j.bjhh.2014.06.002 PMC4318370 25305165

[7] Darbari DS, Nouraie M, Taylor JG, Brugnara C, Castro O, Ballas SK. Alpha-thalassaemia and response to hydroxyurea in sickle cell anaemia. Eur J Haematol. 2014 4;92(4):341–5. 10.1111/ejh.12245 24330217 PMC3962692

[8] Borg J, Phylactides M, Bartsakoulia M, Tafrali C, Lederer C, Felice AE, et al KLF10 gene expression is associated with high fetal hemoglobin levels and with response to hydroxyurea treatment in β-hemoglobinopathy patients. Pharmacogenomics. 2012 10;13(13):1487–500. 10.2217/pgs.12.125 23057549

[9] Sassi H, Bachir D, Habibi A, Astier A, Galactéros F, Hulin A. No effect of CYP450 and P-glycoprotein on hydroxyurea in vitro metabolism. Fundam Clin Pharmacol. 2010 2;24(1):83–90. 10.1111/j.1472-8206.2009.00723.x 19817872

[10] King SB. The nitric oxide producing reactions of hydroxyurea. Curr Med Chem. 2003 3;10(6):437–52. 12570692 10.2174/0929867033368213

[11] Brun M, Bourdoulous S, Couraud PO, Elion J, Krishnamoorthy R, Lapoumeroulie C. Hydroxyurea downregulates endothelin-1 gene expression and upregulates ICAM-1 gene expression in cultured human endothelial cells. Pharmacogenomics J. 2003 1;3(4):215–26. 10.1038/sj.tpj.6500176 12931135

[12] Creary S, Chisolm DJ, O’Brien SH. ENHANCE—(Electronic Hydroxyurea Adherence): A Protocol to Increase Hydroxyurea Adherence in Patients with Sickle Cell Disease. JMIR Res Protoc. 2016 10 3;5(4):e193 10.2196/resprot.6403 27697749 PMC5067359

[13] Vicari P, Barretto de Mello A, Figueiredo MS. Effects of hydroxyurea in a population of Brazilian patients with sickle cell anemia. Am J Hematol. 2005 3;78(3):243–4. 10.1002/ajh.20293 15726590

[14] Yahouédéhou SCMA Adorno EV, da Guarda CC Ndidi US, Carvalho SP Santiago RP, et al Hydroxyurea in the management of sickle cell disease: pharmacogenomics and enzymatic metabolism. Pharmacogenomics J. 12/2018b;18(6):730–9. 10.1038/s41397-018-0045-1 30206297

[15] Agrawal RK, Patel RK, shah V, Nainiwal L, Trivedi B. Hydroxyurea in Sickle Cell Disease: Drug Review. Indian J Hematol Blood Transfus. 2014 6;30(2):91–6. 10.1007/s12288-013-0261-4 24839362 PMC4022916

[16] Quarmyne M-O, Dong W, Theodore R, Anand S, Barry V, Adisa O, et al Hydroxyurea effectiveness in children and adolescents with sickle cell anemia: A large retrospective, population-based cohort: Hydroxyurea Effectiveness in Sickle Cell Anemia. Am J Hematol. 2017 1;92(1):77–81. 10.1002/ajh.24587 27761932 PMC5167640

[17] Sutton M, Bouhassira EE, Nagel RL. Polymerase chain reaction amplification applied to the determination of beta-like globin gene cluster haplotypes. Am J Hematol. 1989 9;32(1):66–9. 2757004 10.1002/ajh.2830320113

[18] Chong SS, Boehm CD, Higgs DR, Cutting GR. Single-tube multiplex-PCR screen for common deletional determinants of alpha-thalassemia. Blood. 2000 1 1;95(1):360–2. 10607725

[19] Silva-Pinto AC, Angulo IL, Brunetta DM, Neves FIR, Bassi SC, Santis GCD, et al Clinical and hematological effects of hydroxyurea therapy in sickle cell patients: a single-center experience in Brazil. Sao Paulo Med J. 2013;131(4):238–43. 10.1590/1516-3180.2013.1314467 24141294 PMC10871833

[20] Torres L de S, Silva DGH da, Junior EB, Almeida EA de, Lobo CL de C, Cançado RD, et al The influence of hydroxyurea on oxidative stress in sickle cell anemia. Rev Bras Hematol E Hemoter. 2012;34(6):421–5. 10.5581/1516-8484.20120106 PMC3545428 23323065

[21] Voskaridou E, Christoulas D, Bilalis A, Plata E, Varvagiannis K, Stamatopoulos G, et al The effect of prolonged administration of hydroxyurea on morbidity and mortality in adult patients with sickle cell syndromes: results of a 17-year, single-center trial (LaSHS). Blood. 2010 3 25;115(12):2354–63. 10.1182/blood-2009-05-221333 19903897

[22] Colombatti R, Palazzi G, Masera N, Notarangelo LD, Bonetti E, Samperi P, et al Hydroxyurea prescription, availability and use for children with sickle cell disease in Italy: Results of a National Multicenter survey. Pediatr Blood Cancer. 2018 2;65(2):e26774. 10.1002/pbc.26774 28868627

[23] Rodgers GP, Dover GJ, Noguchi CT, Schechter AN, Nienhuis AW. Hematologic Responses of Patients with Sickle Cell Disease to Treatment with Hydroxyurea. N Engl J Med. 1990 4 12;322(15):1037–45. 10.1056/NEJM199004123221504 1690857

[24] Chand AR, Xu H, Wells LG, Clair B, Neunert C, Spellman AE, et al Are There True Non-Responders to Hydroxyurea in Sickle Cell Disease? a Multiparameter Analysis. Blood. 2014 12 4;124(21):4073.

[25] Shome DK, Al Ajmi A, Radhi AA, Mansoor EJ, Majed KS. The Effect of Hydroxyurea Therapy in Bahraini Sickle Cell Disease Patients. Indian J Hematol Blood Transfus. 2016 3;32(1):104–9. 10.1007/s12288-015-0529-y 26855516 PMC4733670

[26] Pallis FR, Conran N, Fertrin KY, Olalla Saad ST, Costa FF, Franco-Penteado CF. Hydroxycarbamide reduces eosinophil adhesion and degranulation in sickle cell anaemia patients. Br J Haematol. 2014 1;164(2):286–95. 10.1111/bjh.12628 24383847

[27] Santos FK de S, Maia CN. Patients with sickle cell disease taking hydroxyurea in the Hemocentro Regional de Montes Claros. Rev Bras Hematol E Hemoter. 2010;33(2):105–9. 10.5581/1516-8484.20110029 PMC3520633 23284256

[28] Belini Junior E, Silva DGH, Torres L de S, Okumura JV, Lobo CL de C, Bonini-Domingos CR. Severity of Brazilian sickle cell disease patients: Severity scores and feasibility of the Bayesian network model use. Blood Cells Mol Dis. 2015 4;54(4):321–7. 10.1016/j.bcmd.2015.01.011 25842370

[29] Ji X, Feng Y, Tian H, Meng W, Wang W, Liu N, et al The mechanism of proinflammatory HDL generation in sickle cell disease is linked to cell-free hemoglobin via haptoglobin. PLoS ONE. 2016;11(10):1–19. 10.1371/journal.pone.0164264 PMC5055316 27716784

[30] Lalanne-Mistrih M-D, Connes P, Lamarre Y, Lemonne N, Hardy-Dessources, Tarer V, et al Lipid profiles in French West Indies sickle cell disease cohorts, and their general population. Lipids Health Dis. 2018;17(1):1–8. 10.1186/s12944-017-0646-8 29506549 PMC5836466

[31] Daak AA, Ghebremeskel K, Elbashir MI, Bakhita A, Hassan Z, Crawford MA. Hydroxyurea Therapy Mobilises Arachidonic Acid from Inner Cell Membrane Aminophospholipids in Patients with Homozygous Sickle Cell Disease. J Lipids. 2011;2011:1–8. 10.1155/2011/718014 PMC3173880 21941660

[32] Parhofer KG. Interaction between Glucose and Lipid Metabolism: More than Diabetic Dyslipidemia. Diabetes Metab J. 2015;39(5):353 10.4093/dmj.2015.39.5.353 26566492 PMC4641964

[33] Ragg S, Key M, Rankin F, Hulbert ML. Insights from Comparative Serum Proteomic Profiling of Children with Sickle Cell Disease: The Effect of Hydroxyurea and Genotype on Protein Abundance. Blood. 2016;128(22):1302.27365427

[34] Moreira JA, Laurentino MR, Machado RPG, Barbosa MC, Gonçalves RP, Mota A de M, et al Pattern of hemolysis parameters and association with fetal hemoglobin in sickle cell anemia patients in steady state. Rev Bras Hematol E Hemoter. 2015 5;37(3):167–71. 10.1016/j.bjhh.2015.01.008 PMC4459481 26041418

[35] Iqbal A, Ansari SH, Parveen S, Khan IA, Siddiqui AJ, Musharraf SG. Hydroxyurea Treated β-Thalassemia Children Demonstrate a Shift in Metabolism Towards Healthy Pattern. Sci Rep [Internet]. 2018 12 [cited 2018 Oct 31];8(1). Available from: http://www.nature.com/articles/s41598-018-33540-6 10.1038/s41598-018-33540-6 PMC6182004 30310134

[36] Fitzhugh CD, Wigfall DR, Ware RE. Enalapril and hydroxyurea therapy for children with sickle nephropathy. Pediatr Blood Cancer. 2005 12;45(7):982–5. 10.1002/pbc.20296 15704213

[37] Laurin L-P, Nachman PH, Desai PC, Ataga KI, Derebail VK. Hydroxyurea is associated with lower prevalence of albuminuria in adults with sickle cell disease. Nephrol Dial Transplant. 2014 6 1;29(6):1211–8. 10.1093/ndt/gft295 24084325 PMC4038249

[38] Ware RE, de Montalembert M, Tshilolo L, Abboud MR. Sickle cell disease. The Lancet. 2017 7;390(10091):311–23. 10.1016/S0140-6736(17)30193-9 28159390

[39] Bernaudin F, Arnaud C, Kamdem A, Hau I, Lelong F, Epaud R, et al Biological impact of α genes, β haplotypes, and G6PD activity in sickle cell anemia at baseline and with hydroxyurea. Blood Adv. 2018 3 27;2(6):626–37. 10.1182/bloodadvances.2017014555 29555644 PMC5873235

[40] Adorno EV, Couto FD, Moura Neto JP de, Menezes JF, Rêgo M, Reis MG dos, et al Hemoglobinopathies in newborns from Salvador, Bahia, Northeast Brazil. Cad Saúde Pública. 2005 2;21(1):292–8. 15692663 10.1590/s0102-311x2005000100032

[41] Vasavda N, Badiger S, Rees D, Height S, Howard J, Thein SL. The presence of α-thalassaemia trait blunts the response to hydroxycarbamide in patients with sickle cell disease. Br J Haematol [Internet]. 2008 9 [cited 2018 Jul 8]; Available from: http://doi.wiley.com/10.1111/j.1365-2141.2008.07375.x 10.1111/j.1365-2141.2008.07375.x 18764867

[42] Vasavda N, Woodley C, Allman M, Drašar E, Awogbade M, Howard J, et al Effects of co-existing α-thalassaemia in sickle cell disease on hydroxycarbamide therapy and circulating nucleic acids: Correspondence. Br J Haematol. 2012 4;157(2):249–52. 10.1111/j.1365-2141.2011.08937.x 22082280

[43] Steinberg MH, Hsu H, Nagel RL, Milner PF, Adams JG, Benjamin L, et al Gender and haplotype effects upon hematological manifestations of adult sickle cell anemia. Am J Hematol. 1995 3;48(3):175–81. 7532353 10.1002/ajh.2830480307

[44] Sheehan VA, Luo Z, Flanagan JM, Howard TA, Thompson BW, Wang WC, et al Genetic modifiers of sickle cell anemia in the BABY HUG cohort: influence on laboratory and clinical phenotypes. Am J Hematol. 2013 7;88(7):571–6. 10.1002/ajh.23457 23606168

