# Supplementary material for: Hydroxyurea alters hematological, biochemical and inflammatory biomarkers in Brazilian children with SCA: Investigating associations with βS haplotype and α-thalassemia
Source: PLoS One. 2019 Jul 15;14(7):e0218040. doi: 10.1371/journal.pone.0218040 (PMC6629070; doi:10.1371/journal.pone.0218040)
Supplement: Supplementary file 1 [file pone.0218040.s001.docx]

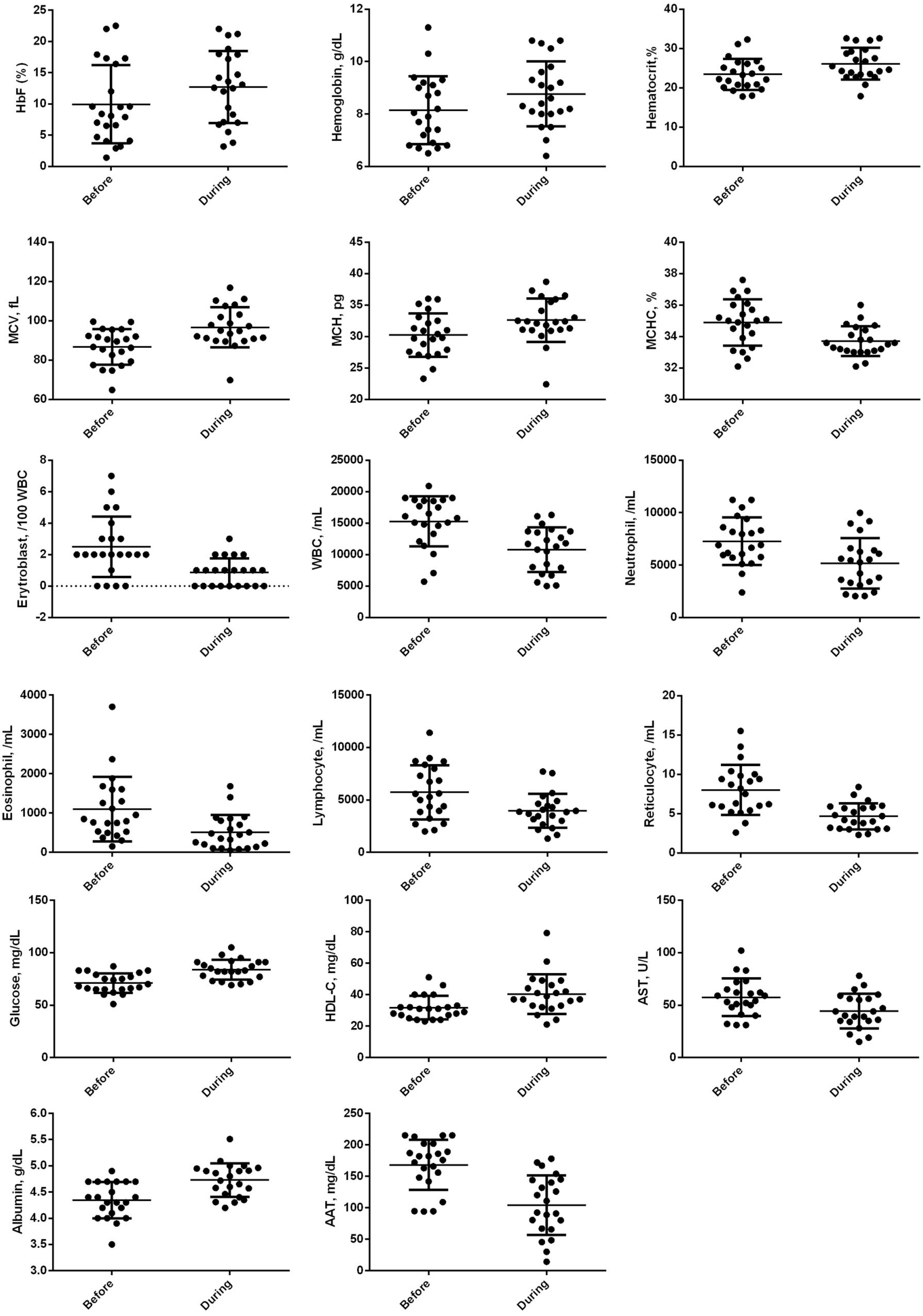
HbF (%)
Hemoglobin, g/dL
Hematocrit,%
MCV, fL
MCH, pg
MCHC, %
Erytroblast, /100 WBC
WBC, /mL
Neutrophil, /mL
Eosinophil, /mL
Lymphocyte, /mL
Reticulocyte, /mL
Glucose, mg/dL
HDL-C, mg/dL
AST, U/L
Albumin, g/dL
AAT, mg/dL
Before
During
